# Supplementary material for: Unveiling the Impact of Gene Presence/Absence Variation in Driving Inter-Individual Sequence Diversity within the CRP-I Gene Family in Mytilus spp
Source: Genes (Basel). 2023 Mar 24;14(4):787. doi: 10.3390/genes14040787 (PMC10138031; doi:10.3390/genes14040787)
Supplement: Supplementary file 1 [file genes-14-00787-s001.zip › Supplementary Figures.pdf]

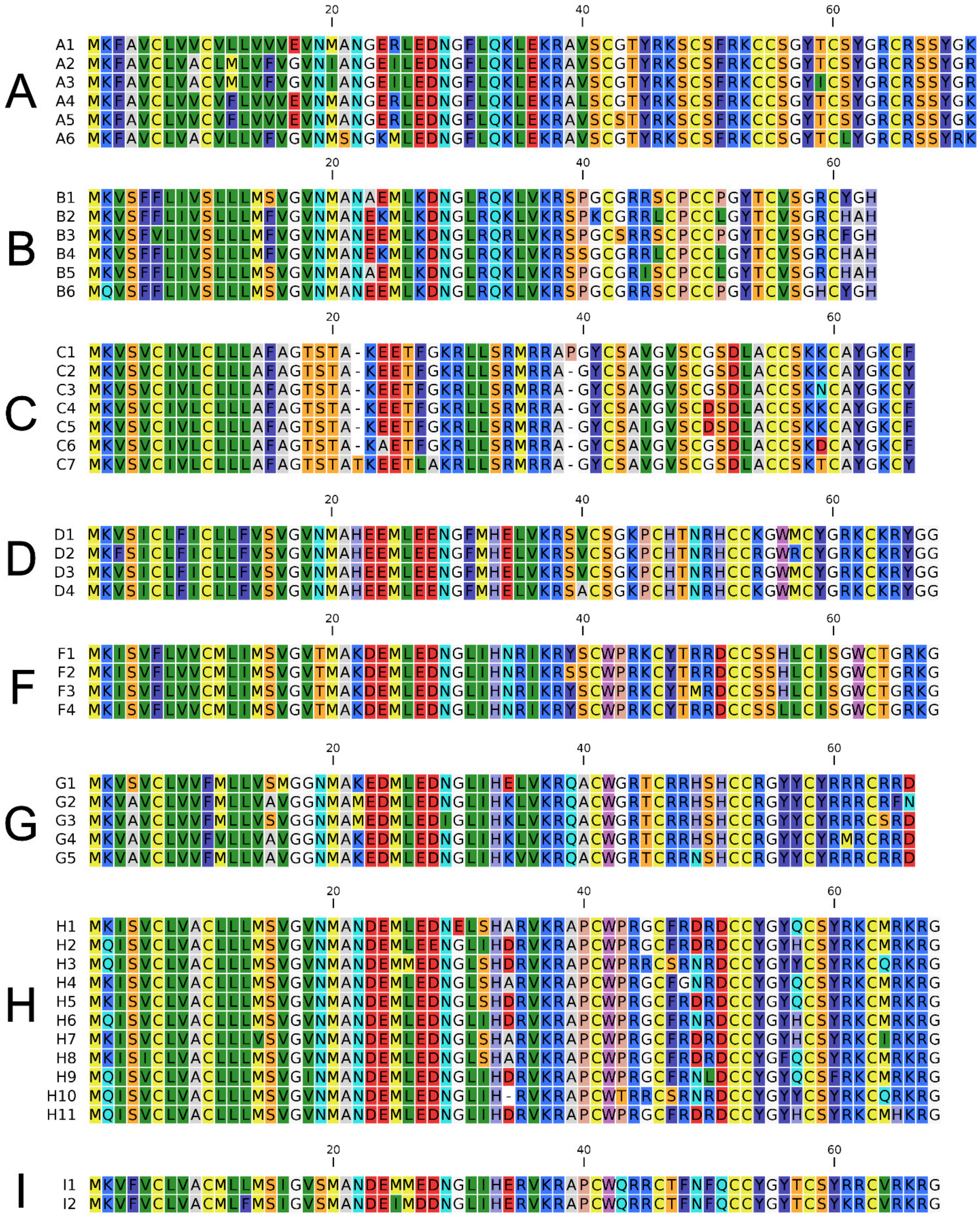

Figure S1: Multiple sequence alignment of the precursor protein sequences belonging to the sCRP-I groups A-I, excluding those including only one member.

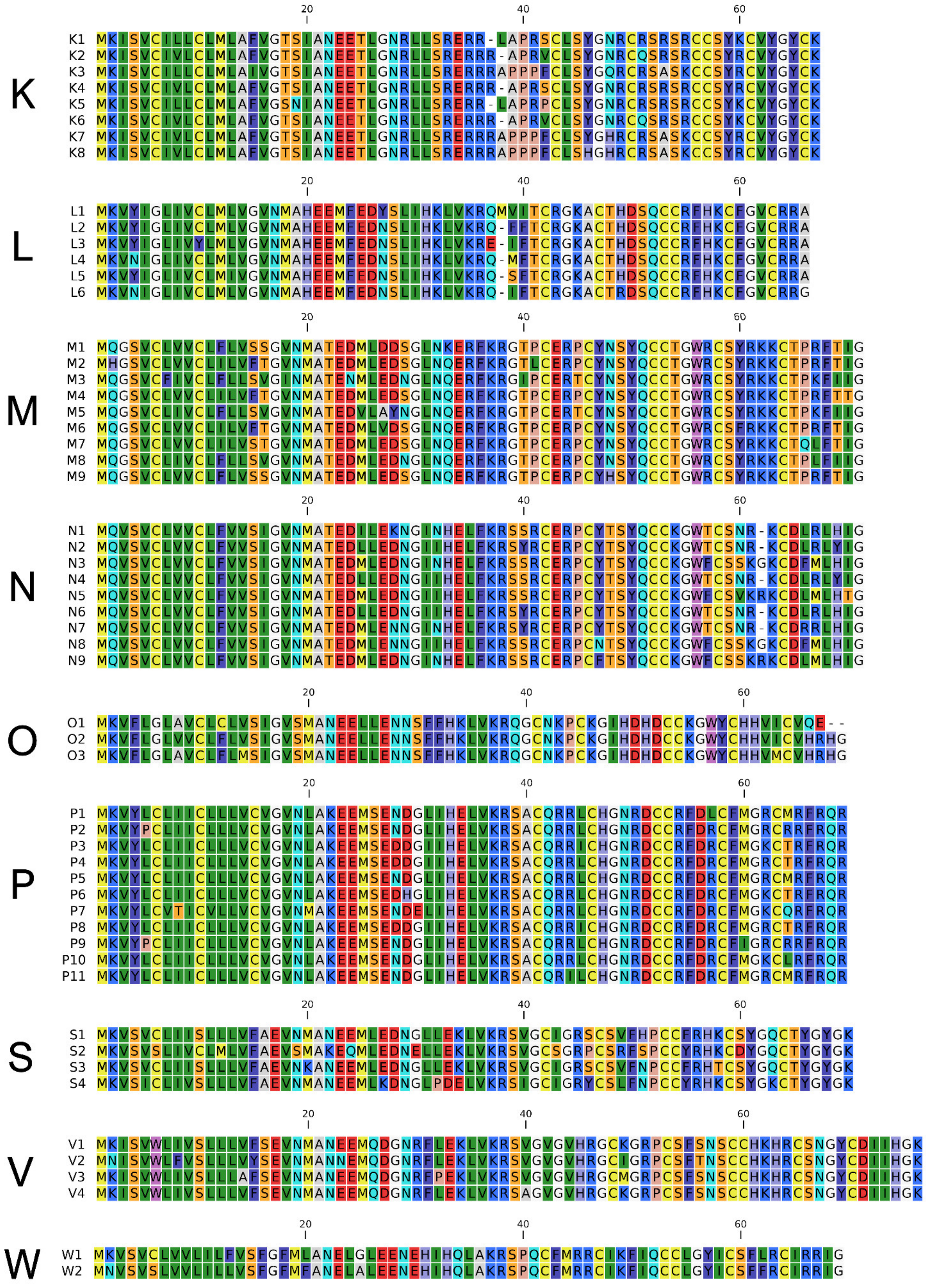

Figure S2: Multiple sequence alignment of the precursor protein sequences belonging to the sCRP-I groups K-W, excluding those including only one member.

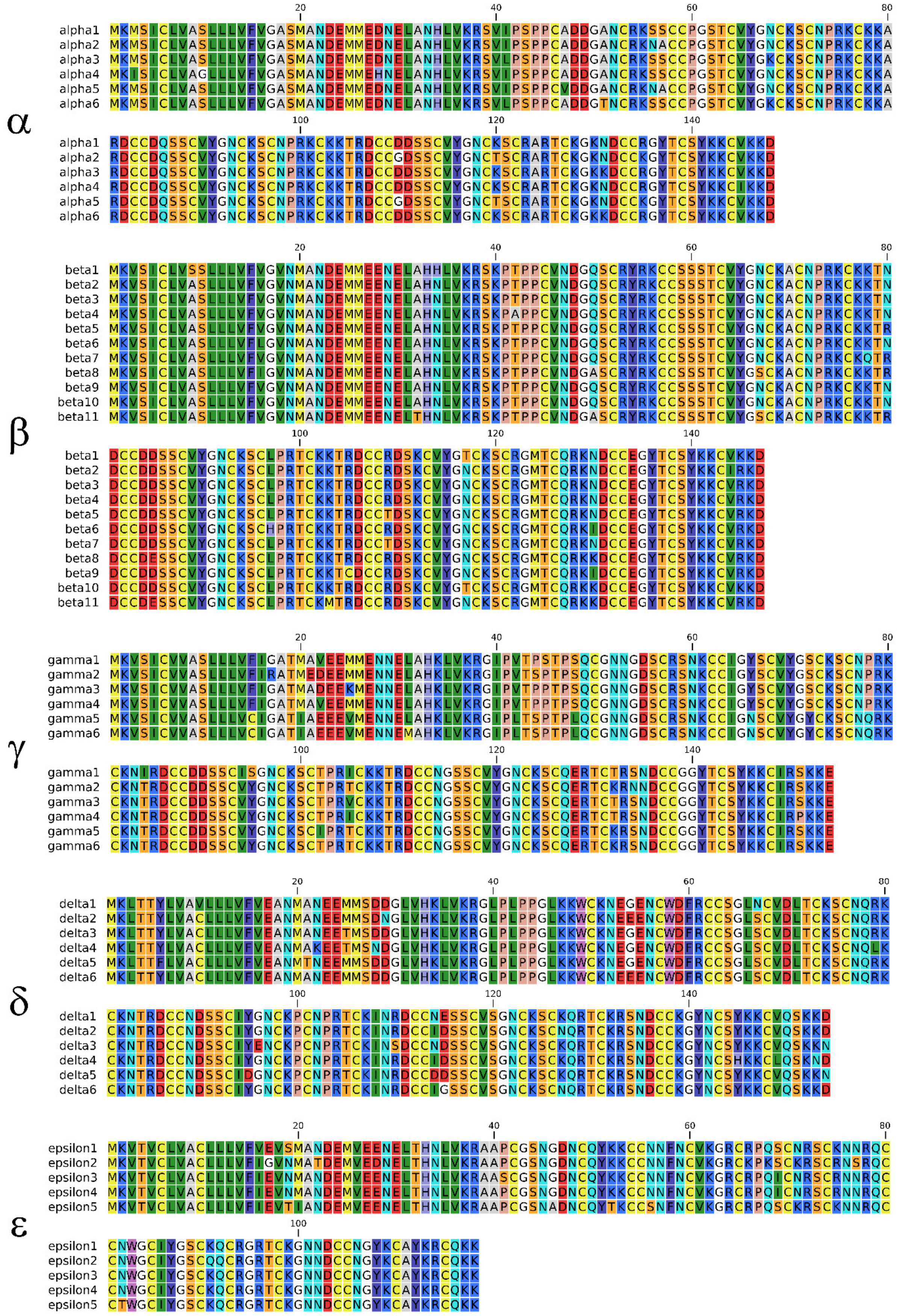

Figure S3: Multiple sequence alignment of the precursor protein sequences belonging to the mCRP-I groups α-ε, excluding those including only one member.

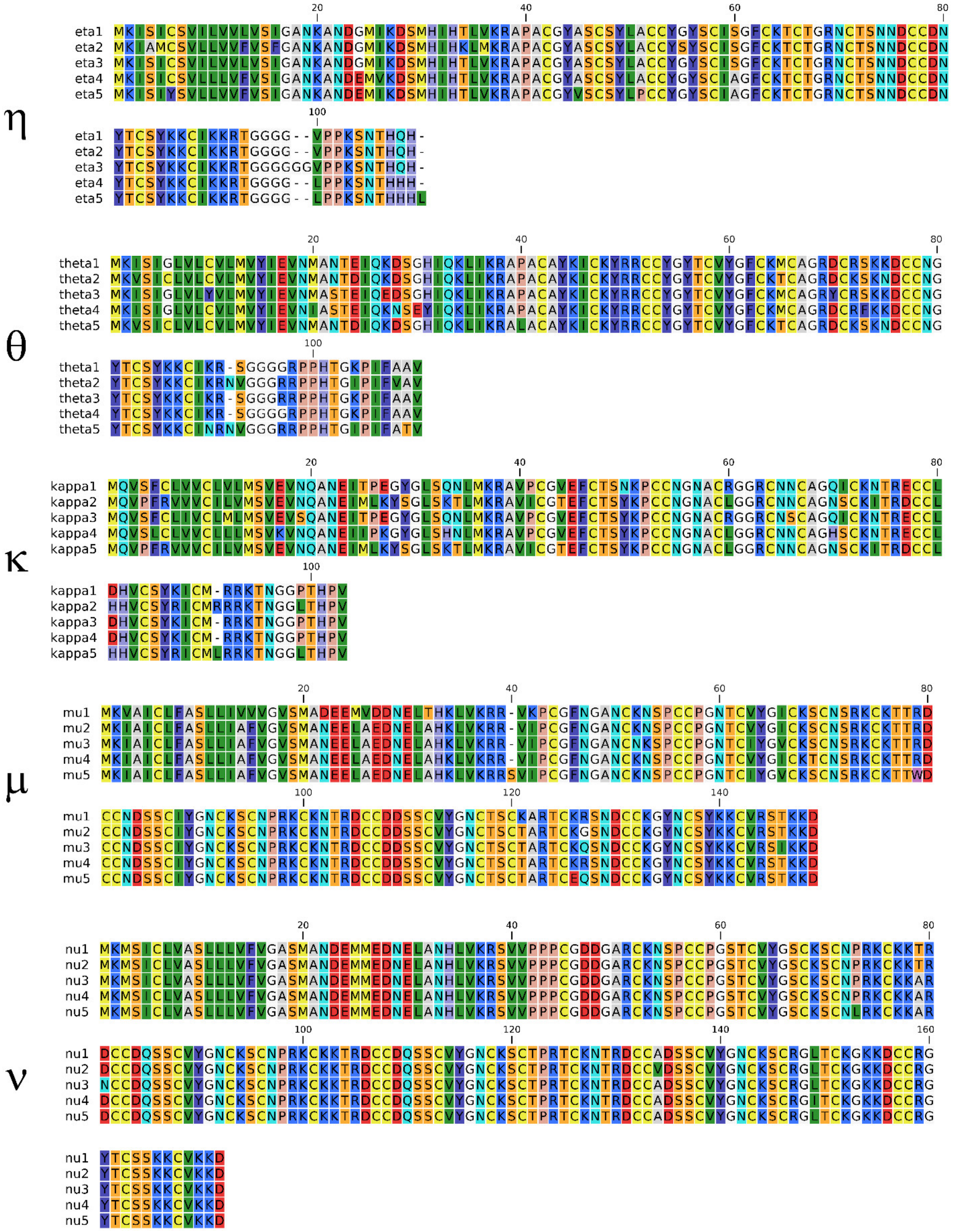

Figure S4: Multiple sequence alignment of the precursor protein sequences belonging to the mCRP-I groups η-ν, excluding those including only one member.

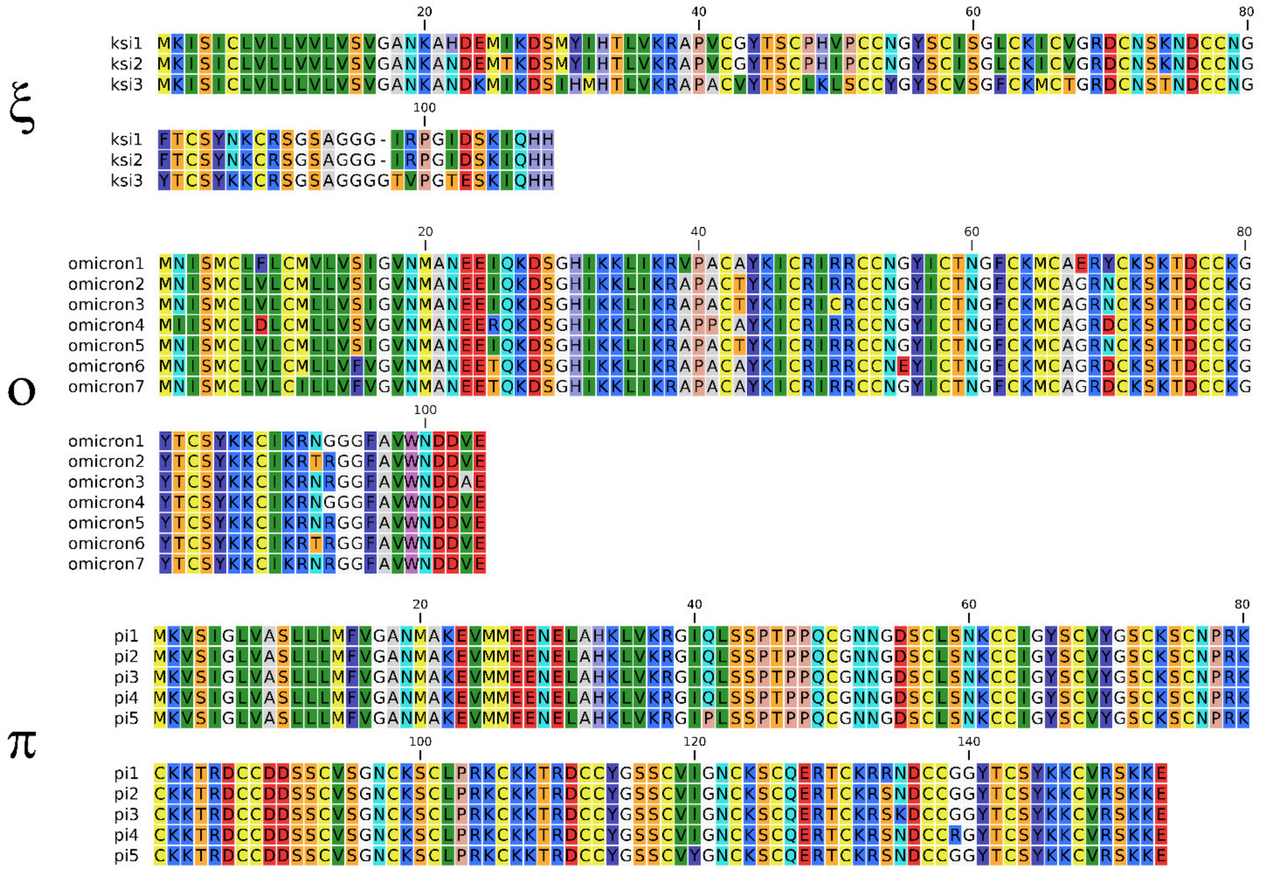

Figure S5: Multiple sequence alignment of the precursor protein sequences belonging to the mCRP-I groups ξ-π, excluding those including only one member.

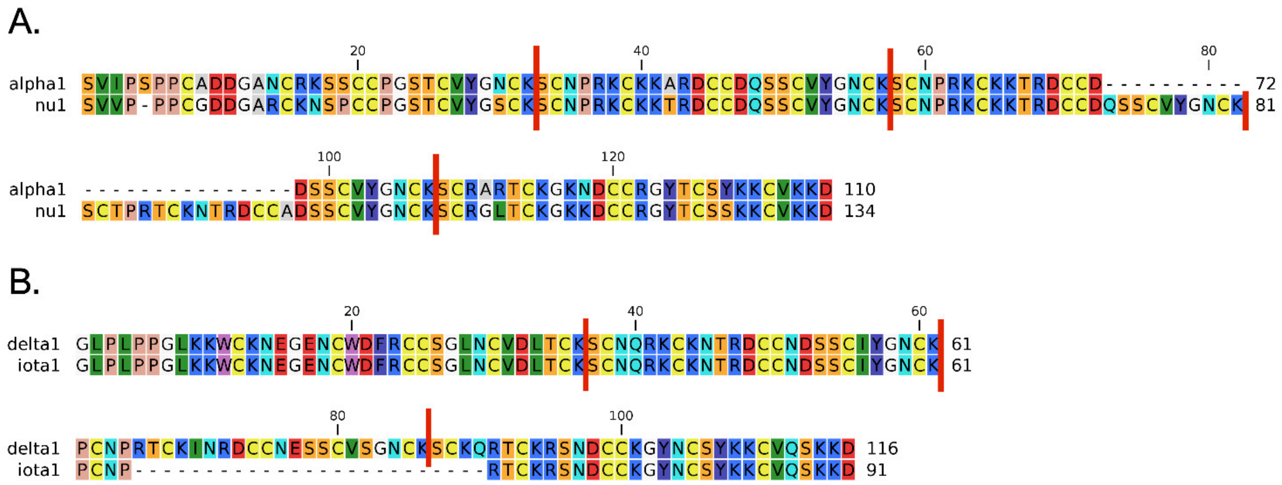

Figure S6: Panel A: alignment between representative members of the mCRP-I subfamilies α and ν. Panel B: alignment between representative members of the mCRP-I subfamilies δ and ι.

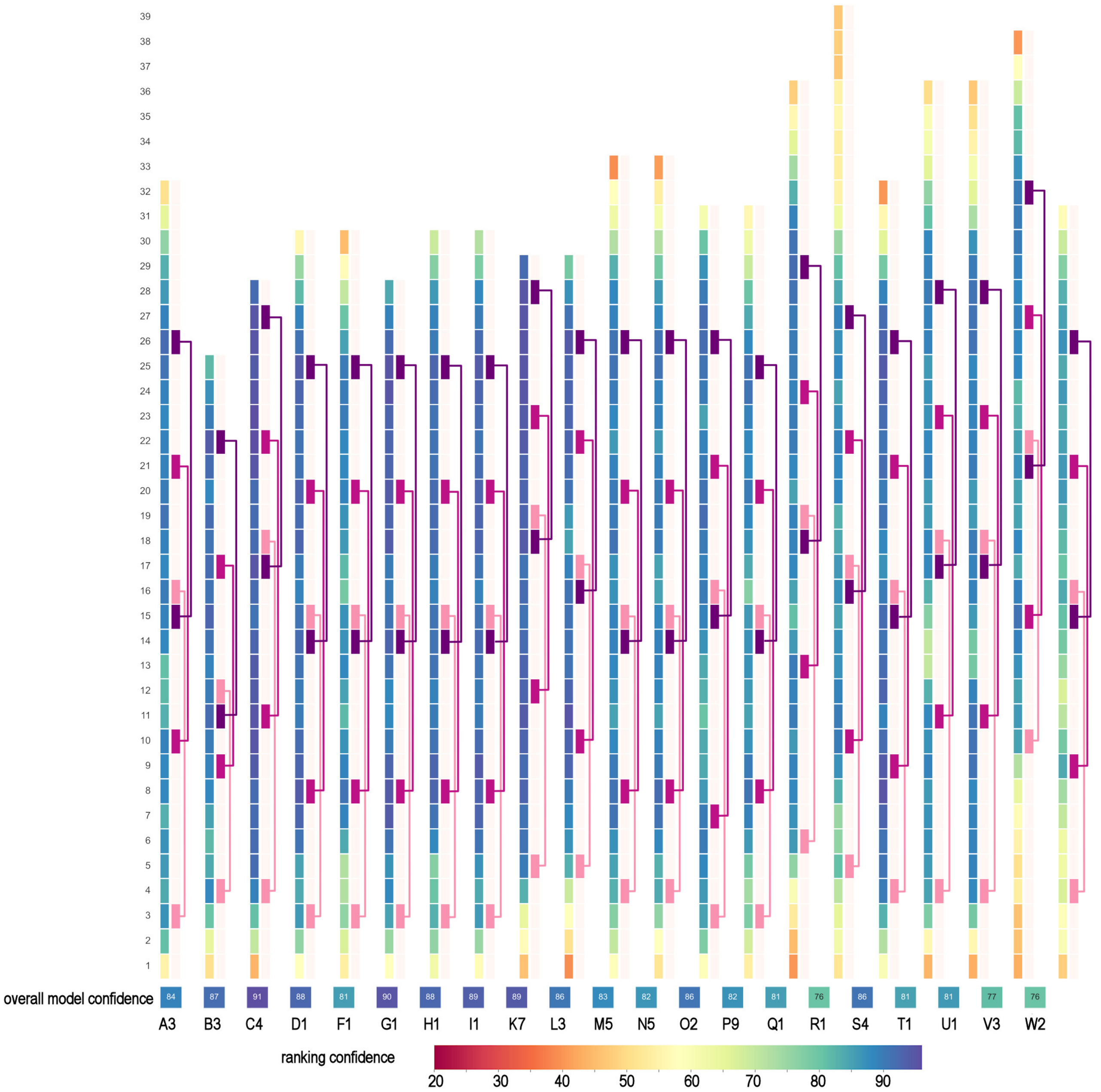

Figure S7: summary of the AlphaFold prediction scores obtained for representative members of the different sCRP-I sequence groups.

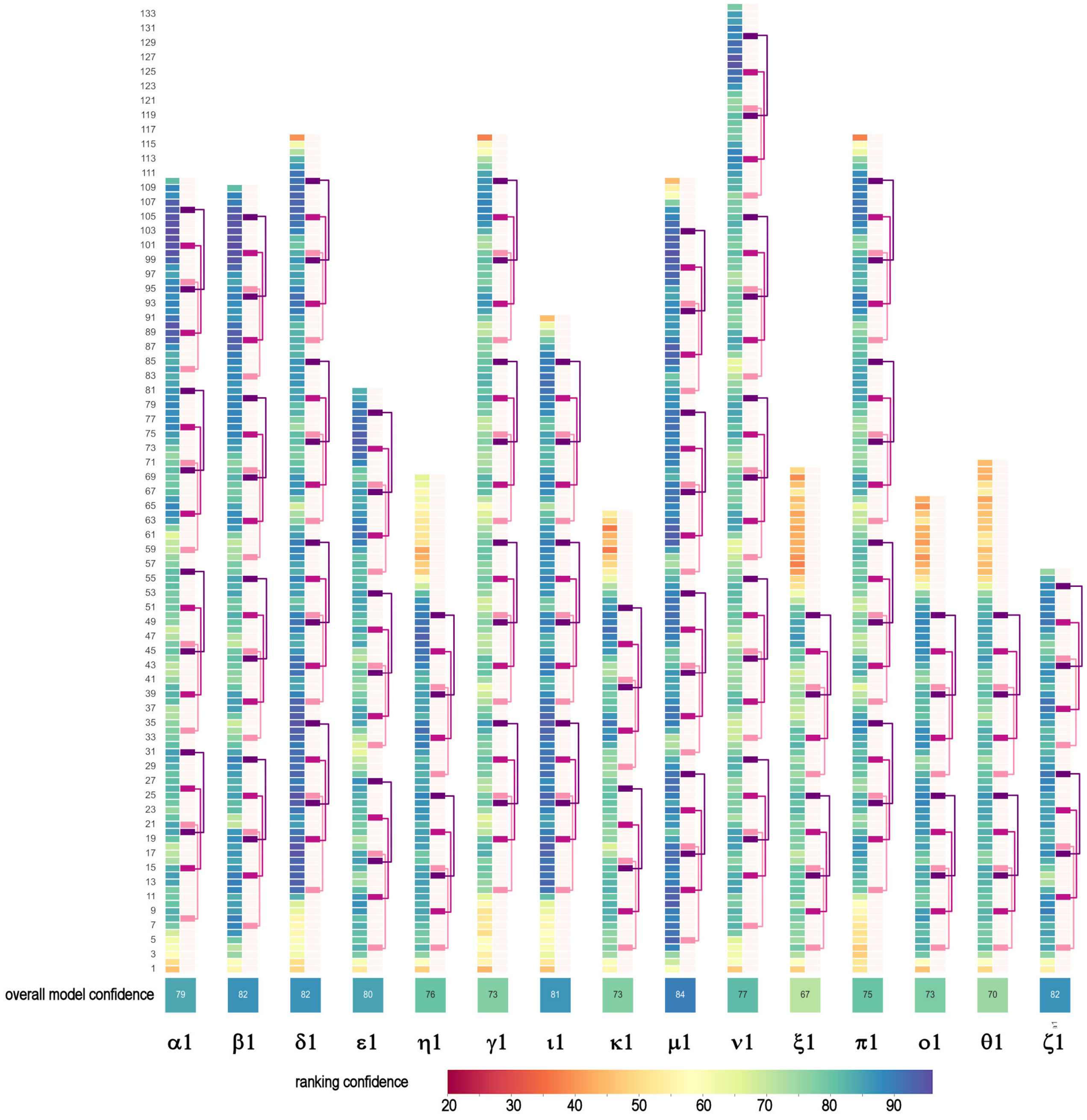

Figure S8: summary of the AlphaFold prediction scores obtained for representative members of the different mCRP-I sequence groups.
