# Supplementary material for: Unveiling the Impact of Gene Presence/Absence Variation in Driving Inter-Individual Sequence Diversity within the CRP-I Gene Family in Mytilus spp
Source: Genes (Basel). 2023 Mar 24;14(4):787. doi: 10.3390/genes14040787 (PMC10138031; doi:10.3390/genes14040787)
Supplement: Supplementary file 1 [file genes-14-00787-s001.zip › Supplementary_Data_Note_1_modified.pdf]

Supplementary Materials Note S1

DNA from the same individual (Anne - SAMN19838403) used to generate the *Mytilus edulis* PEIMed assembly (GCA_019925275.1) was used to generate a PacBio HiFi library. The introgression of *Mytilus trossolus* or *M. galloprovincialis* was ruled out using 12 SNPs previously used to distinguish the member of the *Mytilus* species complex [1]. Sequencing was carried out by the Biotechnology Center and the University of Wisconsin – Madison. In total, 4 flow cells were used to generate the data with the Sequel2 sequencer. Calling of the CSS reads was done by the native PacBio software used by Sequel2. A total of 8,417,094 CSS reads were generated, resulting in coverage ~20X (Genome Size 1.65 Gb). Tissue samples from the same individual were also sent to Dovetail Genomics (Scotts Valley, CA). Dovetail extracted intact nuclei from muscle samples and constructed Omni-C proximity ligation libraries. Omni-C libraries were sequenced using a NovaSeq sequencer (Illumina, San Diego, CA).

We produced a primary/alternative phased assembly as well as haplotype-specific contigs using HiFiasm with the integration of the proximity ligation reads to improve phasing [2]. Putative contaminant sequences from other organisms were removed using blobtools2 [3] (Figure 1). The majority of contigs retained after filtering were putative molluscan sequences. Omni-C reads were mapped to the primary assembly using BWA [4]. The Arima genomics pipeline was used to map and process the mapped reads for scaffolding GitHub - ArimaGenomics/mapping_pipeline: Mapping pipeline for data generated using Arima-HiC). The primary assembly was scaffolded using SALSA2 [5,6] and pin_hic [7]. Metrics for all assemblies can be found in table 1. All scaffolded assemblies had 14 major scaffolds that putatively represent the *M. edulis* chromosomes. The pin_hic assembly had better contiguity than the one produced by SALSA2 (data now shown). Therefore, the pin_hic assembly was used for downstream analysis.

Table 1. Contiguity and completeness metrics for all contig- and scaffold-level assemblies.

Statistics	Contigs				Scaffolds	
	HiFiasm - Primary	HiFiasm - Alternative	HiFiasm - Hap1	HiFiasm - Hap2	PEIMed	HiFiasm - Primary + Pins
# of Contigs	670	23318	905	735	9686	670
# of Scaffolds	NA	NA	NA	NA	1119	347
Total Length (Gb)	1.64	1.36	1.33	1.35	1.65	1.64
Longest Contig (Mb)	84.40	1.30	24.77	22.05	143.80	225.00
N50 (Mb)	16.64	0.20	6.22	6.70	116.50	105.88
L50	30	1807	67	70	7	6
L90	118	13911	226	225	13	14
GC (%)	32.42	32.43	32.42	32.39	32.20	32.36
Complete BUSCOS (%)	95.05	NA	93.73	94.06	90.43	95.05
Fragmented BUSCOS (%)	0.33	NA	0.66	1.98	2.31	0.33
Missing BUSCOS (%)	4.62	NA	5.61	3.96	7.26	4.62
Merqry (C%, CV)	83.59, 50.2	NA	99.0, 50.1*	99.0, 50.1*	83.6, 30	83.50, 50.2

*Combined Haplotypes

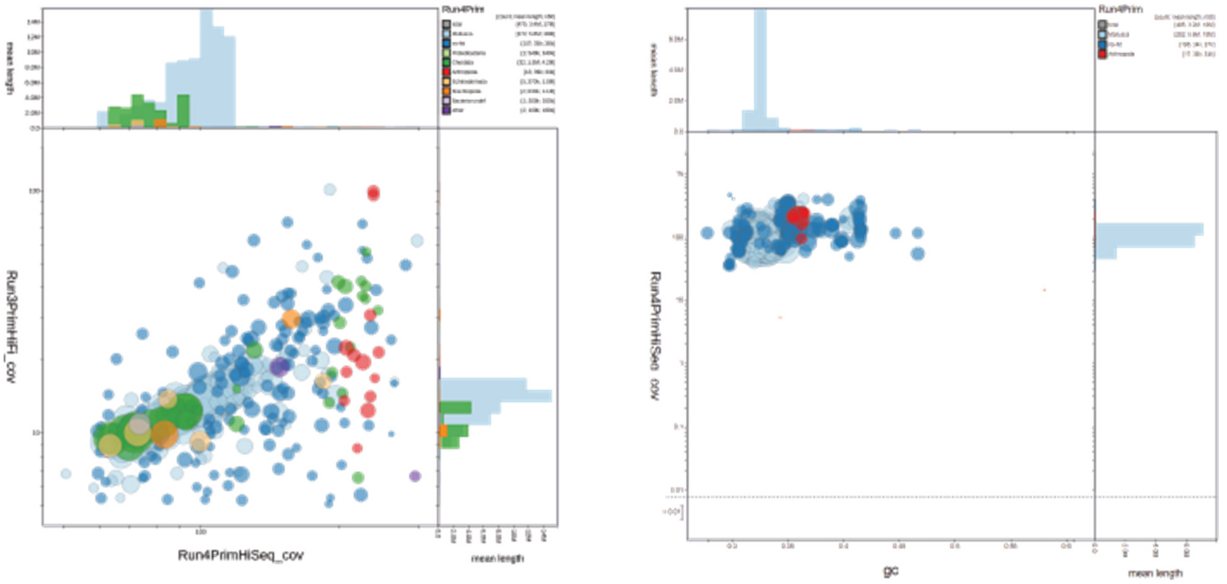

Figure 1. Blobtools2 plots before and after filtering.
